# Are herders protected by their herds? An experimental analysis of zooprophylaxis against the malaria vector *Anopheles arabiensis*

**DOI:** 10.1186/1475-2875-10-68

**Published:** 2011-03-24

**Authors:** Iňaki Tirados, Gabriella Gibson, Stephen Young, Stephen J Torr

**Affiliations:** 1 Natural Resources Institute (NRI), University of Greenwich at Medway, Chatham, UK

## Abstract

**Background:**

The number of *Anopheles arabiensis *(Diptera: Culicidae) and *Anopheles pharoensis *caught by human and cattle baits was investigated experimentally in the Arba Minch district of southern Ethiopia to determine if attraction to humans, indoors or outdoors, was affected by the presence or absence of cattle.

**Methods:**

Field studies were made of the effect of a surrounding ring (10 m radius) of 20 cattle on the numbers of mosquitoes collected by human-baited sampling methods (i) inside or (ii) outside a hut.

**Results:**

The numbers of *An. arabiensis *caught outdoors by a human landing catch (HLC) with or without a ring of cattle were not significantly different (2 × 2 Latin square comparisons: means = 24.8 and 37.2 mosquitoes/night, respectively; n = 12, *P *> 0.22, Tukey HSD), whereas, the numbers of *An. pharoensis *caught were significantly reduced (44%) by a ring of cattle (4.9 vs. 8.7; n = 12, *P *< 0.05). The catch of *An. arabiensis *in human-baited traps (HBT) was 25 times greater than in cattle-baited traps (CBT) (34.0 vs. 1.3, n = 24; *P *< 0.001) whereas, for *An. pharoensis *there was no significant difference. Furthermore, HBT and CBT catches were unaffected by a ring of cattle (4 × 4 Latin square comparison) for either *An. arabiensis *(n = 48; *P *> 0.999) or *An. pharoensis *(n = 48, *P *> 0.870). The HLC catches indoors vs. outdoors were not significantly different for either *An. arabiensis *or *An. pharoensis *(n = 12, *P *> 0.969), but for *An. arabiensis *only, the indoor catch was reduced significantly by 49% when the hut was surrounded by cattle (Tukey HSD, n = 12, *P *> 0.01).

**Conclusions:**

Outdoors, a preponderance of cattle (20:1, cattle:humans) does not provide any material zooprophylactic effect against biting by *An. arabiensis*. For a human indoors, the presence of cattle outdoors nearly halved the catch. Unfortunately, this level of reduction would not have an appreciable impact on malaria incidence in an area with typically > 1 infective bite/person/night. For *An. pharoensis*, cattle significantly reduced the human catch indoors and outdoors, but still only by about half. These results suggest that even for traditional pastoralist communities of East Africa, the presence of large numbers of cattle does not confer effective zooprophylaxis against malaria transmitted by *An. arabiensis *or *An. pharoensis*.

## Background

*Anopheles arabiensis *(Diptera: Culicidae), second only to *An. gambiae s.s*. in its malarial vectorial capacity, is generally described as the 'less anthropophilic' or 'more opportunistic' and more 'exophagic' of the two species, particularly in eastern Africa [1-3]. This suggests that the presence of cattle within human settlements could divert malaria vectors away from humans, thereby reducing malaria transmission by passive zooprophylaxis. As tempting and logical as this proposition sounds, there are two important points to bear in mind.

First, if there is a high underlying entomological inoculation rate (EIR) of, say, 1 infective bite/person/night, then halving this to, say, 0.5 bites/person/night will not provide any material reduction in the incidence of malaria [2].

Second, terms such as anthropophily and exophagy do not describe specific behaviours *per se*, but, rather, are generally descriptive behavioural types inferred from the observed origin of bloodmeals. Drawing inferences about 'host preference' from information about bloodmeals alone can be confounded by the locations where mosquitoes are sampled. For example, in a study of *An. arabiensis *in southern Ethiopia, Tirados *et al *[2] found that in a village where the ratio of cattle:humans was ~ 1:1, the proportions of bloodmeals from humans was 51%. In a nearby cattle camp, however, where the ratio of cattle:humans was ~17:1, the percentage of human bloodmeals was very similar (46%). Thus, *An. arabiensis *seems to be 'opportunistic' in the village, but 'anthropophilic' in the cattle camp. The high proportion of human bloodmeals in the cattle camp begs the question: what is the behavioural mechanism that leads *An. arabiensis *to obtain such a biased feeding pattern?

Attempts to assess the inherent strength of response of *An. arabiensis *to human and cattle odours have generally concluded that this species is significantly more strongly attracted to human than cattle odours. In the study of Tirados *et al *[2], the numbers of *An. arabiensis *females caught in human odour-baited entry traps (OBETs [4]) were ~7.8 times greater than in cattle-baited OBETs, and in response to whole baits, human-landing catches caught about six times more than cattle-baited traps. Tirados *et al *[2] suggested that the paradoxical evidence that a so-called 'opportunistic' species appears to be highly anthropophilic in a cattle camp but not in a village could be explained as an interaction between a preference for feeding on humans and a preference for feeding outdoors. Accordingly, in the village context, the overall cattle:human ratio might be ~ 1:1, but with most humans indoors at night, the effective cattle:human ratio would be much higher. In the cattle camp, humans were always outdoors and hence available to mosquitoes, leading to a higher than expected percentage of meals from them.

A separate study, conducted on *An. arabiensis *in Zimbabwe, investigated possible interactions between behaviours associated with bloodfeeding: (1) the attraction to odour and (2) the 'entry' response associated with pursuit of host indoors [5]. The study focussed explicitly on overcoming the problems of biases in trapping systems by using an arrangement of electrocuting nets to quantify the numbers of mosquitoes attracted to odours of cattle and/or humans dispensed outdoors or indoors. Outdoors, odour from a single human and a single ox attracted similar numbers of *An. arabiensis*. However, if these odours were dispensed indoors, then human odour caught significantly more *An. arabiensis *than ox odour. These results suggest that outdoors, odour from a single human or ox are equally attractive to *An. arabiensis*, but human odour elicits a stronger entry response than does cattle odour. The upshot of these findings is that proportion of bloodmeals from humans will depend on the type (cattle or human) and location (indoors or outdoors) of hosts.

Across the various spatial permutations of cattle and humans, two scenarios might be regarded as being most likely to result in a bias towards feeding from cattle: (1) a large herd of cattle that surrounds a single human outdoors, or (2) the same scenario but with the human indoors. Accordingly, the present study evaluated the impact of cattle barriers on the collection of two malaria vectors, *An. arabiensis *and *An. pharoensis*, from human hosts, indoors and outdoors. The main aim was to discover the degree to which herds might, under ideal circumstances, protect their herders given the underlying blood-feeding behaviour of the mosquito species.

## Methods

### Study site

The study was undertaken between March 2003 and February 2004, in Arba Minch wereda (district) adjacent to Lake Chamo in the Rift Valley of southern Ethiopia. Average annual rainfall totals ~850 mm/year (Ethiopia Meteorological Authority) with the main wet season in April-May and secondary rains in October. The experiments were conducted in Sile, a small village (~10 occupied huts) within Shelle kebele (sub-district) (5.9°N, 37.5°E; ~1600 masl), ~20 km south of the town of Arba Minch. Cattle rearing and rice cultivation are the main economic activities in the area. Irrigated rice fields provide suitable breeding sites for *An. arabiensis *all year round. All experiments described below were run from 19:00 to 7:00 hours each night.

### Identification of species

*Anopheles gambiae s.l. *and *An. pharoensis *mosquitoes were identified morphologically following the keys of Gillies & DeMeillon [6] and Gillies & Coetzee [7]. Previous studies undertaken in the area have shown that >99% of *An. gambiae s.l. *caught at the study site are *An. arabiensis *[8,9].

### Sampling methods

#### Human-landing catch (HLC)

Trained field assistants, sitting on the ground, collected mosquitoes as they landed on the assistants' exposed legs. Collecting teams of two worked over 12 h periods, taking it in turns of 6 hour shifts, except as detailed below. Collectors were provided with malaria prophylaxis under medical supervision, following the guidelines for ethical approval of collaborating institutions (FARM Africa, Addis Ababa; Ministry of Health, Awassa; NRI, University of Greenwich: Project R8214).

#### Cattle-baited/Human-baited trap (CBT/HBT)

This host-baited trap consists of a wooden frame enclosure ~ 2 × 2 × 1 m high, covered with light cotton cloth, except for a gap of ~ 30 cm around the bottom, which was left open overnight to allow mosquitoes to enter. When a bovine was placed in the trap, the gap was closed just before sunrise (~06:00 hours) to retain any blood-fed mosquitoes. This type of trap is recommended for collecting mosquitoes attracted to specific types of bait animal [10]; mosquitoes that feed on the animal during the night generally remain in the enclosure overnight, and are collected in the morning by manual aspiration.

The trap was also operated with a human inside as bait. However, rather than allowing mosquitoes to feed, the human collected mosquitoes as they landed, as with the HLC. Thus, for both the human- and cattle-baited traps, mosquitoes had to have entered the trap through the gap at the bottom of the enclosure. The efficiencies of the two types of trap are unknown, but from the results of Tirados *et al *[2] in Ethiopia, it was expected that the HBTs would be more efficient than the CBTs at entrapping the *An. arabiensis *females that approached the traps. Notwithstanding this difference in trap efficiencies, the traps provided an effective means of measuring the relative numbers of mosquitoes caught in the presence or absence of cattle.

#### Experimental hut

A light-weight, easily transportable hut (1.5 m wide and deep × 2 m tall at the eves) was built so that it could be moved from site to site as dictated by the Latin square study design (see below). Each wall consisted of a double layer of khaki-coloured cotton canvas stretched over a metallic framework; the gaps between the two layers of cloth were filled with straw. A peaked roof, made of straw, rested on the four walls, with open eaves (~ 10 cm wide) around most of the hut. A door (0.5 m high) was left half open during the experiments.

### Experimental treatments

#### Relative catches of a human bait outdoors, with or without a ring of cattle surrounding the bait; Experiments 1 & 2

**Figure 1:**
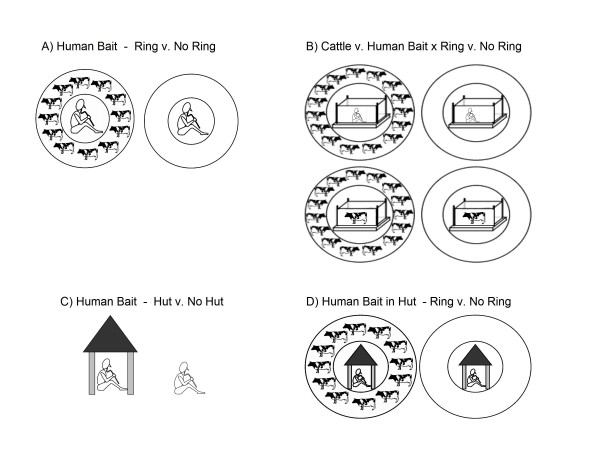
**Arrangements of trapping devices for each experiments**; A) Experiment 1: Comparison of human landing catch, with or without a ring of cattle, B) Experiment 2: Four-way comparison of catches in a human-baited trap or a cattle-baited trap, with or without a ring of cattle, C) Experiment 3: Comparison of indoor or outdoor human-landing catch, D) Experiment 4: Comparison of indoor human-landing catch with or without a ring of cattle around the hut.

To test the effect of the presence of cattle on the numbers of mosquitoes arriving at a human host a HLC was conducted, (1) with or (2) without the presence of a ring of 20 cattle (n = 12 nights; Figure 1A). A circular corral, constructed from a double ring (radius ≈ 10 m) of branches, ensured that the animals maintained their position with respect to the human bait. The cattle were tethered such that there was ~ an animal's length between each of them.

Any change in catch produced by surrounding a human bait with cattle might be due to the diluting effect of having the human as only one of 21 potential hosts (i.e. one human and 20 cattle); studies of the numbers of biting insects attracted to herds of cattle have shown that the *per capita *density of vectors declines with herd size [11]. To examine this possibility, the experiment was repeated, with a CBT or HBT at the centre of the ring. The two types of traps would still have had their respective biases with respect to catching efficiency but, nonetheless, the aim was to test whether the catch from a cattle- or human-baited trap was reduced by surrounding the trap with cattle. Hence, the experiment consisted of four treatments: (1) HBT without a ring of cattle, (2) HBT with a ring of cattle, (3) CBT without a ring of cattle and (4) CBT with a ring of cattle (n = 24 nights; Figure 1B).

#### Relative catches of a human bait inside a hut or outdoors; Experiment 3, and relative catches of a human bait inside a hut with or without a ring of cattle around the hut; Experiment 4

For Experiment 3, a HLC was conducted indoors or outdoors for *An. arabiensis *and *An. pharoensis *(n = 12 nights; Figure 1C). For Experiment 4, a HLC was conducted indoors with or without a ring of cattle outdoors (2 × 2 Latin square, n = 12 nights; Figure 1D). For these experiments, collectors did not alternate shifts, remaining either indoors or outdoors for the full 12 hour period. This protocol was intended to reduce the risk of disturbing the mosquitoes' natural behaviour by opening and shutting the door during the night.

The results of these two experiments were compared to determine whether there was an interaction between the preference of *An. arabiensis *and *An. pharoensis *to feed indoors or outdoors and their respective preference to feed on humans or cattle.

### Experimental design and statistical analyses

For each experiment, treatments were compared in a series of Latin squares of treatments × sites × nights. Each experiment involved different trapping devices and experimental arrangements, so each experiment was analysed separately by analysis of variance (ANOVA). For Experiments 1, 2 and 3, catches (*n*) were transformed to *ln*(*n*+1) before the ANOVA using R [12]. Tukey's HSD multiple range comparisons were used to identify significant differences between specific treatments. For Experiment 4, the *ln *transform produced unsatisfactory distributions of residuals and a square-root transformation was used instead. Means and standard errors given in the text are back-transformed from *ln *mean (or square root mean in the case of Experiment (4) and standard errors are derived from the ANOVA residual mean square.

## Results

### Do malaria vectors fly past cattle in response to a human bait?

**Figure 2:**
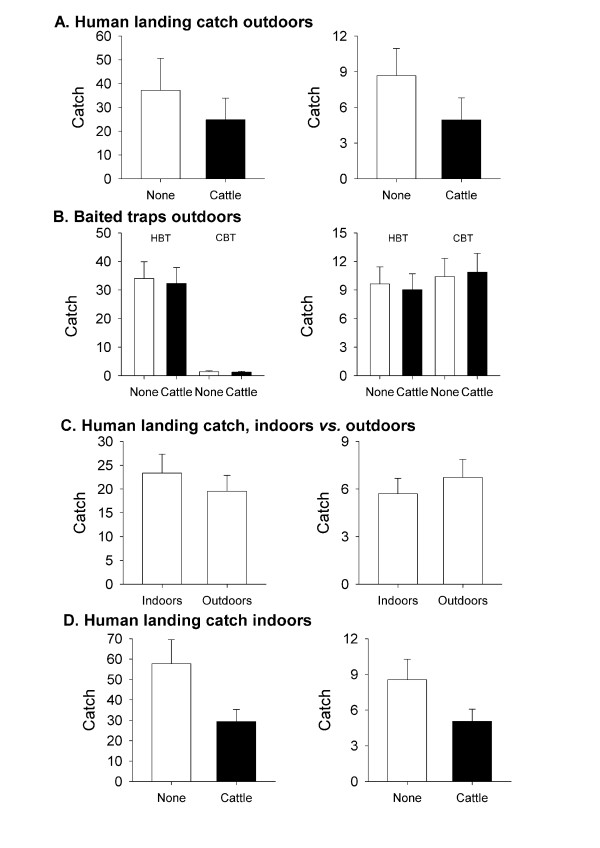
**Histograms of mean catches for each experiment**; A) Experiment 1: Comparison of human landing catch, with or without a ring of cattle, B) Experiment 2: Four-way comparison of catches in a human-baited trap or a cattle-baited trap, with or without a ring of cattle, C) Experiment 3: Comparison of indoor or outdoor human-landing catch, D) Experiment 4: Comparison of indoor human-landing catch with or without a ring of cattle around the hut (*Anopheles arabiensis *plots on left; *Anopheles pharoensis *plots on right). Means and standard errors are back-transformed from ln mean (or sqrt mean in the case of Experiment 4) and standard errors are derived from the ANOVA residual mean square.

Overall, for Experiment 1, the catch of *An. arabiensis *in HLCs was reduced with cattle present, but the effect was not significant (means = 24.8 and 37.2 mosquitoes/night, respectively; n = 12 replicates; F = 1.7; *P *> 0.22, Figure 2A), whereas for *An. pharoensis*, the catch was significantly less (44%) with a ring of cattle than without (4.9 vs. 8.7 mosquitoes/night; n = 12, *P *< 0.05, Figure 2A).

For Experiment 2, the catches of *An. arabiensis *in HBTs and CBTs, respectively, were similarly unaffected by the addition of a ring of cattle surrounding the baited trap (ANOVA, n = 48; F_1,184 _= 0.902; *P *> 0.764, Figure 2B). The catches of *An. arabiensis *in HBTs, whether surrounded or not by cattle, was > 25 times greater than in CBTs (*P *< 0.001 for trap:spp, Figure 2B), whereas for *An. pharoensis*, there was no significant difference between the numbers caught in HBTs and CBTs, irrespective of whether or not they were surrounded by cattle (*P *> 0.869 for trap:spp, Figure 2B).

Again, it is striking that, in spite of the availability of so many cattle, a significantly greater number of *An. arabiensis *flew through the cattle ring to a human-baited trap than to a cattle-baited trap, whereas similar number of *An. pharoensis *were caught in the two types of trap, irrespective of the presence of the cattle ring.

Overall, these results demonstrate that *An. arabiensis *flew though the ring of cattle because (1) they were responding primarily to the human stimuli and/or (2) the diverting effect of cattle was counterbalanced by an increase in numbers of mosquitoes attracted to the area. Either way, the 'herd' did not afford much protection to the 'herder'.

### Is there an interaction between hut entry behaviour and host preference?

There was no significant difference between HLCs indoors or outdoors when no cattle were present (Experiment 3) across species (*F*_***1,44 ***_= 0.0015; *P *> 0.969, Figure 2C), or for *An. arabiensis *alone (*P *> 0.854 Figure 2C). Thus, without competing hosts present, both species appear to enter huts that contain a human host readily.

Overall, for Experiment 4, the ring of cattle around the hut led to a significant reduction in indoor HLCs (F_1,44 _= 11.64; *P *< 0.001). The *An. arabiensis *catch was reduced by 49% (*P *< 0.01, Tukey HSD). The *An. pharoensis *catch was reduced by 41% (*P *> 0.1983), but this was not statistically significant, possibly because the sample sizes were small; overall, *An. pharoensis *catches were only 16% of *An. arabiensis *catches (F = 197, *P *< 0.001).

The results suggest that for *An. arabiensis *there is an interaction between host accessibility and the entry response; cattle did not have a significant effect on a human bait catch outdoors but did have a significant effect when the human was indoors (Figure 2A,B,D). Nonetheless, while there was a statistically significant reduction, the effect was only ~ 50%.

## Discussion

The most striking result of this study is the demonstration that outdoors, catches from humans and human-baited traps were not significantly reduced by surrounding the bait with a ring of 20 cattle (Figure 2B,D). Even when a human was indoors, a ring of cattle reduced the catch significantly but the reduction was a relatively modest halving in catch.

The present study only measured the numbers of mosquitoes landing on a human or caught in a trap. And although the former is an important epidemiological parameter, neither provides information on the behavioural basis of the present results. It seems likely that placing large numbers of cattle in the vicinity of a human would have increased the numbers of mosquitoes attracted to the vicinity [5,13]. Assuming, conservatively, that a ten-fold increase in the number of hosts doubled the numbers attracted to the vicinity [14], then the observation that there was no increase in the catch suggests that more than half the mosquitoes attracted fed on the cattle. Nonetheless, even in this competing miasma of cattle odour, kairomones from humans attracted *An. arabiensis *in densities that were not materially different from the number recruited by a human alone.

This apparent high degree of responsiveness to human host cues is more often associated with the sibling species *An. gambiae s.s*. Although the pattern of responses of *An. pharoensis *was broadly similar to that of *An. arabiensis*, a notable exception is the difference in catch sizes of CBTs. The inference is either that human and cattle host cues were equally attractive for *An. pharoensis*, but not for *An. arabiensis *(Figure 2B) or that there are gross differences between the two species in the efficiency with which they are caught by CBTs and HBTs. Resolving these alternative explanations will require further studies.

A second striking result is the demonstration that, when no other hosts were present, HLCs for *An. arabiensis *females were similar when the human host was indoors or outdoors. Indeed, the HLC was greater, albeit not significantly so, when the collector was indoors. These results contrast with those of Torr *et al *[5] from Zimbabwe, who found that not all *An. arabiensis *attracted to a source of human odour dispensed from within a hut or a trap, entered. In the present study, it was only when cattle surrounded the hut that the indoor catch was reduced. Presumably, the probability of a mosquito entering a hut is the result of odours eliciting a 'hut-entry response' competing against other olfactory cues - e.g. cattle and humans - from outdoor sources. This suggests a subtle, but important nuance in its reputation for being 'exophagic'; *An. arabiensis *females may indeed feed outdoors frequently, but it appears this behaviour may be enhanced when easily accessible hosts are available outdoors, and not, as is often supposed, due to an inherent preference for feeding outdoors *per se*.

### Practical implications

The present results have implications for the likely benefits of passive zooprophylaxis. In cattle camps, where cattle and humans remain outdoors throughout the night, *An. arabiensis *in southern Ethiopia is so strongly anthropophilic [2] that herdsmen surrounded by cattle are not likely to be protected by the cattle from being bitten to any significant degree. The present results indicate that surrounding dwellings with cattle may offer some degree of protection to people in villages when they are indoors. However, at times of year when *An. arabiensis *is abundant, the inoculation rate in this part of Ethiopia can be ~1 infective bite/night [2] and in these circumstances halving the biting rate would not confer any zooprophylactic benefit. Moreover, this modest level of protection may be offset by the greater numbers of mosquitoes sustained in the area by the cattle.

The implications for the use of insecticide-treated cattle (ITC) as a means of reducing *An. arabiensis *populations are, however, generally more promising. It has been proposed that populations of *An. arabiensis *could be reduced/controlled in much the same way as cattle are used to control tsetse populations [15]. Habtewold *et al *[9] has shown that cattle treated with 1% deltamethrin pour-on formulation are lethal to *An. arabiensis*. Taken together with the results of Tirados *et al *[2] the present study shows that even though *An. arabiensis *is highly anthropophilic, it frequently lands on cattle, which means that wherever cattle are present, there is a good chance the mosquitoes will be killed by ITC-treated animals outdoors, particularly where the cattle:human ratio is high.

### Theoretical implications

The differential response of *An. arabiensis *to human host cues outdoors and indoors supports previous indications [5] that there is an interaction between attraction to a particular type of host and hut entry behaviour. The 'entry response' might be inhibited by the presence of alternative or competing host cues (Figure 2D). Thus, these results support the hypothesis that the 'opportunism' of *An. arabiensis *as evidenced by bloodmeal analyses might be the result of an interaction between anthropophily and exophagy. *Anopheles arabiensis *may be no less strongly attracted than *An gambiae s.s. *by human host cues, but they are more inhibited by house entry when alternative hosts are available, effectively leading to higher proportions of cattle:human bloodfeeding in villages than in cattle camps.

### Anthropophily/zoophily paradox

The paradox described by Tirados *et al *[2] that *An. arabiensis *is anthropophilic enough to transmit malaria efficiently and yet zoophilic enough to obtain more than half its bloodmeals from cattle, is resolved to some degree by the results of the present study, which has shown that although *An. arabiensis *is fundamentally highly anthropophilic, (they bypass cattle to obtain a human blood meal outdoors), they have a tendency to be diverted away from human hosts indoors if there are cattle nearby. These findings are consistent with the observation of Tirados *et al *[2] that 46% of bloodmeals were of human origin in an area where the cattle: human ratio was 17:1. *Anopheles arabiensis *apparently 'seeks out' humans, even when there is a surplus of cattle available.

The behavioural basis of this anthropophilic behaviour is still not clear. There is evidence that differential attraction to particular host odours is only part of the story and that behaviour at the host may play a role. Habtewold *et al *[9] found that > 20% of *An. arabiensis *landing on a calf did not feed successfully over an observation period of 10 min, with ~ 35% staying on the animal for < 1 min. Some of these may have settled to feed elsewhere on the animal, or possibly flown on in response to air-borne cues. Several studies have shown a relatively high rate of mixed meals in *An. arabiensis *(13% mixture from different cattle within a herd of 3 cattle [9], 37% mixture of human and cattle blood in an area with a cattle: human ratio of 17:1 [2]). Taken together with the observation that ~ 35% of *An. arabiensis *that land on an ox stay for < 1 min, a general picture emerges that a sizeable proportion of *An. arabiensis *may land on several hosts in a night, taking partial bloodmeals from more than one host, and that the relatively high proportion of human bloodmeals found where cattle predominate, may be due in part at least, to a greater probability for *An. arabiensis *to stay and feed if it lands on a human than on an ox. Furthermore, if indeed *An. arabiensis *hops between hosts, then insecticide treatment of cattle (ITC) would be a far more efficient way of using cattle to reduce malaria transmission than passive prophylaxis: ITC prevents mosquitoes from moving on to the next, potentially human, host and they are killed before they have a chance to bloodfeed and reproduce. Zooprophylaxis affords neither of these benefits.

## Conclusions

Surrounding a human with a large number of cattle reduced the numbers of *An. arabiensis *and *An. pharoensis *landing on the human by ~30-50%. Such a reduction is unlikely to afford any material passive zooprophylaxis against malaria in areas of high endemicity in southern Ethiopia, but the finding provides further evidence that treating cattle with insecticides could be an effective means of reducing transmission. The finding that the reduction in landing catches was greater when the human was indoors suggests that the probability of hut entry is affected by the presence of competing cues produced by hosts outside the hut.

Given the high degree of variability in the bloodfeeding and resting behaviour of *An. arabiensis *recorded across its distribution [1], protection from malaria afforded herders by their herds is likely to vary accordingly.

## Competing interests

The authors declare that they have no competing interests.

## Authors' contributions

IT, SJT and GG conceived the study. IT carried out the experiments. SY, GG and SJT analysed the data. GG and SJT drafted the paper. All authors read and approved the final manuscript.
